# Dynamic Obstacle Avoidance for Unmanned Underwater Vehicles Based on an Improved Velocity Obstacle Method

**DOI:** 10.3390/s17122742

**Published:** 2017-11-27

**Authors:** Wei Zhang, Shilin Wei, Yanbin Teng, Jianku Zhang, Xiufang Wang, Zheping Yan

**Affiliations:** Marine Assembly and Automatic Technology Institute, College of Automation, Harbin Engineering University; Harbin 150001, China; dawizw@163.com (W.Z.); tengyanbin@hrbeu.edu.cn (Y.T.); zhangjianku@hrbeu.edu.cn (J.Z.); wangxiuf1990@163.com (X.W.); yanzheping@hrbeu.edu.cn (Z.Y.)

**Keywords:** unmanned underwater vehicle, velocity obstacle method, dynamic collision avoidance, forward-looking sonar

## Abstract

In view of a dynamic obstacle environment with motion uncertainty, we present a dynamic collision avoidance method based on the collision risk assessment and improved velocity obstacle method. First, through the fusion optimization of forward-looking sonar data, the redundancy of the data is reduced and the position, size and velocity information of the obstacles are obtained, which can provide an accurate decision-making basis for next-step collision avoidance. Second, according to minimum meeting time and the minimum distance between the obstacle and unmanned underwater vehicle (UUV), this paper establishes the collision risk assessment model, and screens key obstacles to avoid collision. Finally, the optimization objective function is established based on the improved velocity obstacle method, and a UUV motion characteristic is used to calculate the reachable velocity sets. The optimal collision speed of UUV is searched in velocity space. The corresponding heading and speed commands are calculated, and outputted to the motion control module. The above is the complete dynamic obstacle avoidance process. The simulation results show that the proposed method can obtain a better collision avoidance effect in the dynamic environment, and has good adaptability to the unknown dynamic environment.

## 1. Introduction

The unmanned underwater vehicle (UUV), as an intelligent device for long-term remote underwater navigation, must carry a variety of sensors and special equipment for performing particular missions and tasks [1,2,3]. Among them, the forward-looking sonar in the UUV search and obstacle avoidance process has an unparalleled importance. How to effectively use the front view sonar to obtain information and achieve rapid obstacle avoidance has become a focus of attention [4,5,6].

In the ocean environment, apart from static obstacles, UUV also faces the threat of dynamic obstacles such as underwater floating objects and the other underwater vehicles. The state of known dynamic obstacles can be predicted easily; however, dynamic obstacles in the underwater environment are sudden and unpredictable. For the safety of navigation, the collision avoidance of unknown dynamic obstacles is gaining more and more attention.

The common collision avoidance methods include artificial aperture method (APF) [7,8,9], dynamic window method (DWA) [10] and behavior method [11]. They have strong adaptability to local environments and rely on limited sensor information to avoid collisions and have high efficiency. As the external environment information and its own state has uncertain and partially unknown characteristics, people also often use the intelligent control algorithm in the collision avoidance process, including the fuzzy system, the expert system and deep reinforcement learning and so on. Conti [7] proposes an innovative decentralized approach for cooperative mobile manipulation of Intervention-Autonomous Underwater Vehicles (I-AUVs) based on a different use of potential field method. Navigation and control problems are reduced to the evaluation of the distance vector among the vehicles, object and obstacles. Subramanian [8] proposes a multi-point potential field (MPPF) Autonomous Underwater Vehicle (AUV) three-dimensional path-planning method that introduces the direction of search in the potential field, and does not need to calculate the potential gradient. In the paper [9], the proposal makes use of the Artificial Potential Field (APF) method with a Bacterial Evolutionary Algorithm (BEA) to obtain an enhanced flexible path planner method in environments with static and dynamic obstacles, taking all the advantages of using the APF method. Inara [10] applies DWA to autonomous navigation of AUV in a three-dimensional environment. The paper [11] proposes a technique for avoiding obstacles based on the behavioral structure. In this technique, when a mobile robot gets close to an obstacle, while moving toward its target, a rotational potential field is applied to lead the mobile robot to avoid the obstacle, without locating local minimum positions. Aim [12] presents type-2 fuzzy ontology-based semantic knowledge (T2FOBSK). The distance to closest point of approach (DCPA), time to closest point of approach (TCPA) and variation of compass degree (VCD) are used to calculate the degree of collision risk between AUVs and obstacles. A concise deep reinforcement learning obstacle avoidance (CDRLOA) algorithm is proposed with the powerful deep Q-network architecture to overcome the usability issue caused by the complicated control law in the traditional analytic approach [13]. In order to avoid dynamic obstacles in a timely manner during manufacturing tasks performed by manipulators, D. Han [14] proposes a novel method based on distance calculation and discrete detection. The paper [15] presents the design and implementation of sampling-based path-planning methods for a AUV to avoid collision with commercial aircraft and other moving obstacles. The velocity obstacle (VO) is a conical space that is generated in the robot’s velocity space, and also is a set of velocity vectors. As long as the current velocity vector is outside of the VO, the robot will not collide with an obstacle at any time in the future. Based on this feature, we can conduct real-time motion planning by combining the technology of graphics and the method of optimal control [16]. Ivan R. Bertaska [17] designed a planner that combines a local search based on the VO concept with a global, lattice-based search for a dynamically feasible trajectory. In the paper [18], the proposed approach is based on velocity obstacles (VO) method, which generated a cone-shaped obstacle in the velocity space. However, one of the important premises of VO is to assume that the moving obstacle only does the uniform linear motion; it cannot apply to the situation that the motion path of obstructions is arbitrary. Shiller presents the nonlinear velocity obstacle (NLVO) method, which will consider the shape, size and path curvature of an obstacle [19]. Based the above results, the article [20] introduces the time of avoiding the collision in order to estimate the distance among obstacles, and uses the A* algorithm to search the optimal velocity. On account of the shape, size and velocity uncertainty of obstacles, Kluge raises the probability velocity obstacle (PVO), which takes into account the error of barrier shape and speed. Jamie [21] describes a method of mixed reciprocal velocity obstacle, and it is used for coordinated avoidance of multiple sports. In the paper [22], a safety collision avoidance method is depicted for unmanned surface vehicles (USV) in a dynamic situation. It combines VO with the International Regulations for Preventing Collisions at Sea (COLREGS) and based on the maritime rules of crossover, transcendence and encounter, and sets up search rules for the best speed of collision avoidance.

The method of velocity obstacle also exists all kinds of problems.

1. The uncertainty of obstacle motion

There is a variety of uncertainties for a moving obstacle, such as position, size and speed. An important prerequisite for the velocity obstacle is that the barrier speed will stay constant in the decision cycle, otherwise it will collide. NLVO pays attention to the obstacle of nonlinear motion, but needs to know the trajectory of an obstacle, and it does not apply to unknown obstacles [19]. PVO takes the error of obstacle shape and speed into account, but demands to undertake multiple integral operations, so that a large computation is necessary [20]. The direct expansion method is the most common. Based on the error of the radius, position and speed, it will expand VO in the maximum expansion circle [22,23]. Van proposes that obstacles can be expanded in the maximum velocity [24]. The worst scenario is considered and the result is relatively conservative. The contribution of the paper [25] is that a method was demonstrated to extract the collision cones of circular and non-circular objects using a laser sensor.

2. The opportunity uncertainty of collision avoidance

It is vitally significant for collision avoidance to determine the right opportunities in the course of UUV dynamic anti-collision. Through calculating velocity obstacle, we can easily weigh up UUV collision with the barriers and UUV safe navigation collision speed, but cannot gain the time and distance of obstacle avoidance, so that the opportunities of anti-collision are not clear. Paper [16] only takes the distance of collision avoidance into account. Paper [26] thinks over the time of anti-collision. In paper [27], the multi-step space that a robot can reach in the time of collision avoidance is presented, and the time factor and the distance factor are considered synthetically. Meanwhile, if there are numerous obstacles near the UUV, the UUV will have very few velocity candidates. In paper [28], a method for choosing optimal velocity components using the concept of pass-time and vertical clearance is proposed for the efficient movement of a robot.

3. Processing of large static obstacles

When using the velocity obstacle method to make dynamic avoidance decisions, the current literatures deal less with large static obstacles. The moving obstacles are only considered in few workspaces [16,21]. Although paper [29] pays attention to both static obstacles and moving barriers, it is assumed that the position and shape of static obstacles are known or that the size is small. If the large static obstacles that present in a dynamic environment are expanded directly, it may be overinflated, so that the optimal path cannot be found. In view of this situation, the minimum safe distance method is presented in paper [29]. A double-detection window of different size is used in article [28], and the static obstacles only are handled in the smaller window. This way may reduce the problem of excessive expansion of obstacles, but will lead to obtaining incomplete information about barriers.

In view of the above questions, a dynamic obstacle avoidance system based on improved velocity obstacle is proposed in this paper. An outline of the paper is as follows: some background on the method of velocity obstacle is presented in Section 2. Section 3 details the dynamic collision obstacle method based on improved velocity obstacles, and includes the treatment of obstacles, the method of decision and so on. Extensive simulation results and a practical example of this method are provided in Section 4. Section 5 raises some discuss. Finally, concluding remarks are made in Section 6.

## 2. Preliminaries

### 2.1. Environmental Modeling

**Figure 1:**
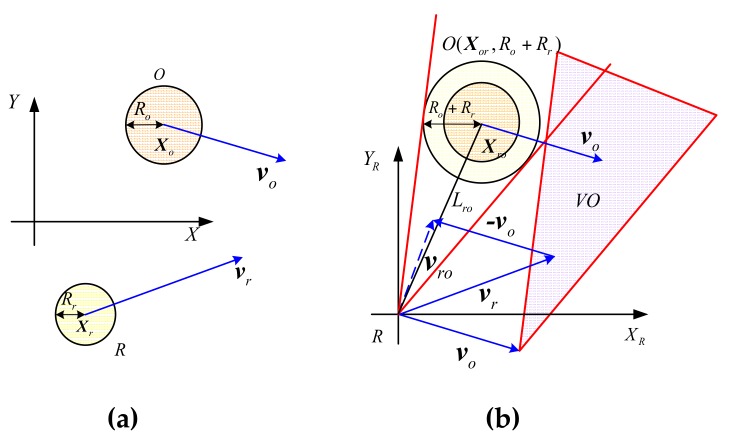
Collision cone and velocity obstacle: (**a**) The relationship between UUV and an obstacle in *X-Y* coordinate system; (**b**) The relationship between UUV and an obstacle in speed obstacle avoidance system.

In Figure 1, R is defined as UUV, and O is a moving obstacle. The UUV and obstacles are expanded into the round moving body, and their radii are $R_{r}$ and $R_{o}$ respectively. In the global Cartesian coordinate system *X*-*Y*, UUV can be expressed as $R(t)=(X_{r}(t),v_{r}(t))$, and $X_{r}(t)=(x_{r}(t),y_{r}(t))\in {\mathbb{R}}^{2}$ is center position of UUV, $v_{r}(t)$ is speed vector of UUV. Meanwhile, moving obstacles can be expressed as $O\left(t\right)=(X_{o}(t),v_{o}(t))$, and $X_{o}(t)=(x_{o}(t),y_{o}(t))\in {\mathbb{R}}^{2}$ is obstacle center position, $v_{o}(t)$ is speed vector of the obstacles. t sets as the current time, ΔT sets as the decision-making period, and the speed of UUV and all moving obstacles are assumed to be constant in the decision-making period.

A relative Cartesian coordinate system *X_R_*-*Y_R_* is established by regarding current position of UUV as the origin, and its axis direction is same as the global coordinate, shown in Figure 1b. The UUV is assumed to be a particle; moving obstacles are expanded to generate correspondingly the set of configuration obstacles $O(X_{or},R_{or})$, whose radius is $R_{or}=R_{o}+R_{r}$. The relative position of an obstacle in a local coordinate is $X_{or}(t)=X_{o}(t)-X_{r}(t)$. If we consider $O(X_{or},R_{or})$ as the static obstacle, the relative speed of between UUV and a dynamic obstacle is $v_{ro}(t)=v_{r}(t)-v_{o}(t)$. Then UUV speed can be expressed as $v_{ro}(t)$, and the problem of dynamic collision avoidance can be transformed into static collision avoidance. In other words, collision avoidance decision of UUV and a moving obstacle O, that the state of motion is, respectively, $\left(X_{r}(t),v_{r}(t)\right)$ and $O(X_{or},R_{or})$, is equal to collision avoidance decision of UUV with the speed of $v_{ro}(t)$ and the static obstacle.

The condition that UUV collides with the static obstacles $O(X_{or},R_{or})$ is $\lambda (v_{ro})\cap O(X_{or},R_{or})\neq \emptyset$, and the ray from the origin along the direction of velocity v is expressed by λ(v)={tv|t>0}. In the velocity space, the collision cone (CC) that consists of a set of the velocity vectors is defined by

(1) $$CC=\{v_{ro}|\lambda (v_{ro})\cap O(X_{or},R_{or})\neq \emptyset \}$$

At the same time, the velocity obstacle (VO) is represented as

(2) $$VO=\{v|\exists t>0,t(v-v_{o})\in CC\}$$

So (2) can be equivalent to

(3) $$VO=CC⊕v_{o}$$

Where ⊕ denotes the Minkowski vector sum operation, and VO defines the set of the speed $v_{r}$ that UUV may collide with an obstacle O.

In conclusion, the condition that UUV collides with a single obstacle O can be shown by

(4) $$X_{r}\notin O(X_{or},R_{or})\wedge v_{r}\notin VO$$

Meanwhile, we use the following formula to denote the condition that UUV collides with multiple obstacles.

(5) $$X_{r}\notin \overset{n}{\underset{i=1}{\cup }}O(X_{o_{i}r},R_{o_{i}r})\wedge v_{r}\notin VO_{r}$$

### 2.2. Process Analysis of Speed Collision Avoidance

**Figure 2:**
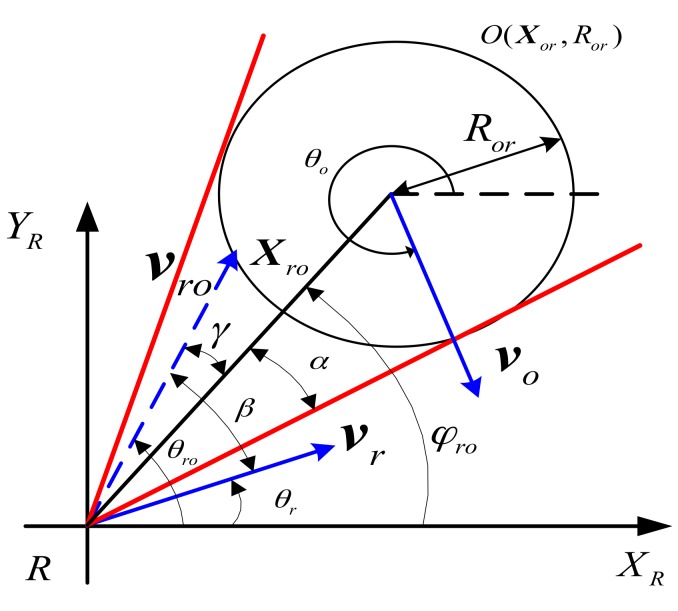
Process analysis of speed collision avoidance.

As shown in Figure 2, in the relative Cartesian coordinate system, velocity component $V_{s}$ that relative velocity $v_{ro}$ along the sight $X_{ro}$, and velocity component $V_{\theta }$ that $v_{ro}$ perpendicular to $X_{ro}$ are expressed, respectively, as

(6) $$\begin{array}{l} V_{s}=v_{r}\cos(\theta_{r}-\varphi_{ro})-v_{o}\cos(\theta_{o}-\varphi_{ro})\\ V_{\theta }=v_{r}\sin(\theta_{r}-\varphi_{ro})-v_{o}\sin(\theta_{o}-\varphi_{ro}) \end{array}$$

Where the intersection angle of $v_{ro}$ and $X_{ro}$ is collision angle γ, and the linear distance between the obstacle and UUV is $\left\|X_{ro}\right\|$, and the radius of the obstacle is $R_{or}$. The safety angle is $\alpha =\arcsin(\frac{R_{or}}{\left\|X_{ro}\right\|})$. By comparing the relative relationship of collision angle γ and safety angle α, the condition which UUV collides with the obstacle can be obtained.

(7) γ≤α

When the collision angle is bigger than the safety angle, as γ>α, UUV will not collide with obstacles, so that UUV navigation with current speed is safe.

Collision angle γ can be expressed as

(8) $$\tan(\gamma )=\frac{V_{\theta }}{V_{s}}$$

tan(γ) is a function about $v_{r},v_{o},\theta_{r},\theta_{o}$, as $\tan(\gamma )=f(v_{r},\theta_{r},v_{o},\theta_{o})$, γ=arctan(f). A derivation of γ is

(9) $$d\gamma =\frac{df}{1+f^{2}}$$

β is the intersection angle of $v_{ro}$ and $v_{r}$. Further, we can turn the Equation (9) into a differential equation on the derivation of γ.

(10) $$\Delta \gamma =\frac{-\sin(\beta )\Delta v_{r}+v_{r}\cos(\beta )\Delta \theta_{r}}{v_{ro}}$$

From the collision conditions of Equation (10), Δγ can be changed to make sure UUV safety collision avoidance by adjusting $\Delta \theta_{r}$ and $\Delta v_{r}$.

## 3. Dynamic Collision Avoidance Based on Improved Speed Obstacle Method

In this section, we present a theoretical framework for UUV dynamic collision avoidance based on an improved speed obstacle method. It can be separated into obstacle information processing, collision risk assessment, key obstruction screening and collision avoidance decision.

### 3.1. Obstacle Information Processing

#### 3.1.1. Obstacle Property Detection and Classification

The task that UUV must complete is to reach the destination from a given starting position and avoid all kinds of unknown obstacles in the course of the movement. Therefore, the received obstacle information must be processed. In this paper, this processing can be presented in three steps as follows.

1. Obstacle property detection and classification

Combined with the forward-looking sonar and working characteristics of UUV, we will employ the moving target detection method presented in paper [24]. This method can identify the obstacle properties in the UUV working environment and distinguish between static obstacles and dynamic obstacles.

2. The division of ranging points

Obstacles can be expressed as several isolated sets of ranging points in Scanning charts of the forward-looking sonar. The distances between the points in the point set are closer, compared with the distance between point sets. The current data of ranging points can be divided based on this property. As long as the distance between adjacent points is less than a certain threshold, they are considered to belong to the same obstacle set.

3. Match and classification of obstacle sets

The division of ranging points is not enough to acquire the movement properties of the obstacles, so comparison of the difference between the adjacent time grid maps is necessary, and it can detect the movement of obstacles (movement or static).

For two sets of obstacle points for adjacent moments $\Omega_{m_{1}(n)},\Omega_{m_{2}(n-1)}$, we defined the non-coincidence function $G_{as}=1-\frac{S_{\Omega_{m_{1}}(n)}\cap S_{\Omega_{m_{2}}(n-1)}}{S_{\Omega_{m_{1}}(n)}\cup S_{\Omega_{m_{2}}(n-1)}}$, then combined with the centroid distance of the two obstacle sets $F_{as}=\left\|\left({\bar{\rho }}_{m_{1}},{\bar{\theta }}_{m_{1}}),({\bar{\rho }}_{m_{2}},{\bar{\theta }}_{m_{2}}\right)\right\|$, and associative estimation function $J_{as}[\Omega_{m_{1}}(n),\Omega_{m_{2}}(n-1)]$ is built.

(11) $$J_{as}[\Omega_{m_{1}}(n),\Omega_{m_{2}}(n-1)]=\frac{f_{F}}{F_{as}}+\frac{f_{G}}{G_{as}}$$

Where $f_{F}$ and $f_{G}$ are coefficients. S means that the two barrier sets contain the number of grid points. The symbol ∪ indicates the common grid number between the two sets, and ∩ denotes the overlapping grid number. When the distance from the center of mass is closer; the non-coincidence degree is smaller; the value of the associated function is greater, and the possibility that two barriers is the same barrier is bigger. On account of different values of data association functions, the type of obstruction can be determined by setting the associated thresholds $\tau_{same},\tau_{move},\tau_{static}$($\tau_{same}<\tau_{move}<\tau_{static}$).

#### 3.1.2. Static Obstacle Clustering Based on K-Means Algorithm

The static obstacles present some lone grid points $\Omega_{o}=\{X_{c}^{1},X_{c}^{2},\ldots ,X_{c}^{n}\}$ in ROGM (Rolling Occupancy Grid Map), and the distribution of point sets will not undergo dramatic changes in the adjacent moment. Therefore, using the K-means clustering algorithm [30], the set of static obstacle points in ROGM can be partitioned with several smaller static stumbling blocks. These little static obstacles can be thought of as moving obstacles at zero speed, which then can avoid barriers by means of the speed avoidance method. Besides, we define the maximum clustering radius $R_{o}^{\max}$ ($R_{o}^{\max}=60\;m$ in this paper) to prevent large static obstacles from expanding too much. When the radius of the static obstacle $R_{o}>R_{o}^{\max}$, we will adopt the secondary K-means clustering method to divide it.

#### 3.1.3. Motion Parameters Estimation and Uncertainly Analysis of Dynamic Obstacle

1. Motion parameters estimation of dynamic obstacle

The perceptual error of the previewing sonar can cause discontinuity in some of the motor parameters, and the obstacle chain, built by the match and classification method, may be broken. In order to make up for the missing information of the obstacle, the least-squares method is used to predict the location information and the motion parameters in the future. We define the magnitude $v_{o}(n)$ and direction $\theta_{o}(n)$ of dynamic obstacles velocity, acceleration $a_{o}(n)$ and angular velocity $\omega_{o}(n)$ respectively in *n* time. Therefore, the prediction velocity and direction of the moving obstacle in the n+1 moment.

(12) $$\begin{array}{l} {\tilde{v}}_{o}(n+1)=v_{o}(n)+a_{o}(n)\Delta T\\ {\tilde{\theta }}_{o}(n+1)=\theta_{o}(n)+\omega_{o}(n)\Delta T \end{array}$$

2. Uncertainly analysis of dynamic obstacle

The acceleration information of the obstacle reflects the random movement of the obstacle. We assume that $a_{o}$ and $\omega_{o}$ satisfy the normal distribution, $a_{o}\sim N(\mu_{a},\sigma_{a}^{2})$, $\omega_{o}\sim N(\mu_{\omega },\sigma_{\omega }^{2})$, where $\mu_{a},\;\sigma_{a}^{2}$ is the mean and variance, respectively, of $a_{o}$, and $\mu_{\omega },\;\sigma_{\omega }^{2}$ is the mean and variance, respectively, of $\omega_{o}$. $\Delta v_{o},\Delta \theta_{o}$ is the greatest deviation, respectively, from $v_{o}(n)$ and $\theta_{o}(n)$. According of the literature [31], the radius of the uncertainty circle is $\Delta R_{o}$.

(13) $$\Delta R_{o}=\Delta T\sqrt{{\left[v_{o}(n)+\Delta v_{o}\right]}^{2}+v_{o}{\left(n\right)}^{2}-2[v_{o}(n)+\Delta v_{o}]v_{o}(n)\cos(\Delta \theta_{o})}$$

where $\Delta v_{o}=\Delta T\max(\mu_{a}+3\sigma_{a},\mu_{a}-3\sigma_{a}),\Delta \theta_{o}=\Delta T\max(\mu_{\omega }+3\sigma_{\omega },\mu_{\omega }-3\sigma_{\omega })$.

Therefore, the expansion circle with radius $R_{o}+\Delta R_{o}$ is the largest area of the barrier at the next moment.

### 3.2. Hazard Assessment of Collision

**Figure 3:**
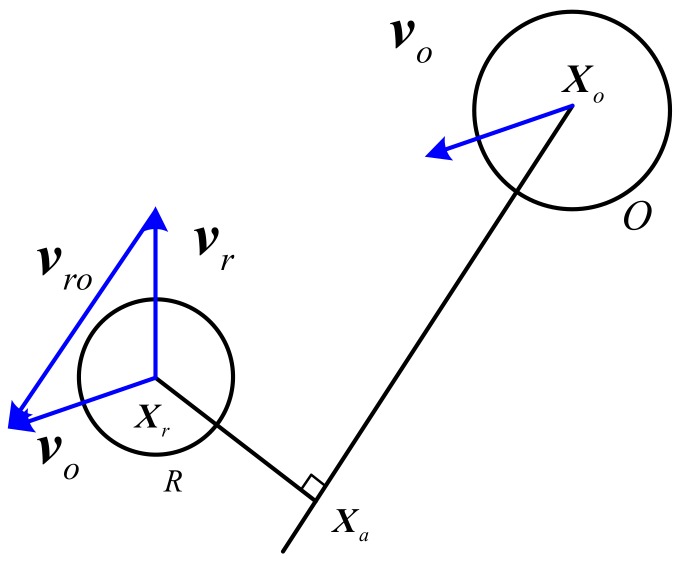
The sketch map of DCPA and TCPA.

UUV and obstacles are considered to be particle as shown in Figure 3. UUV is the point $X_{r}$ with speed $v_{r}$, and moving obstacle is the point $X_{o}$ with speed $v_{o}$. $v_{ro}$ is the relative speed of the moving obstacle and the UUV. Drawing parallel line $X_{o}X_{a}$ parallel to $v_{ro}$ through the $X_{o}$ point, and $X_{r}X_{a}$ perpendicular to $X_{o}X_{a}$ at $X_{a}$ through the $X_{r}$ point. Then $X_{a}$ is the meeting point CPA [32].

According to closest encounter point CPA, the minimum encounter time $T_{CPA}$ of obstacles and UUV is as follows [24]

(14) $$T_{CPA}=\{\begin{array}{cc} 0 & \left\|v_{o}(t)-v_{r}(t)\right\|\leq \varepsilon \\ \frac{\left(X_{o}(t)-X_{r}(t)).(v_{r}(t)-v_{o}(t)\right)}{{\left\|v_{o}(t)-v_{r}(t)\right\|}^{2}} & \mathrm{else} \end{array}$$

After calculating minimum encounter time, the minimum encounter distance of UUV and obstacles can be expressed as

(15) $$D_{CPA}=\left\|\left(X_{o}(t)+v_{o}(t).T_{CPA})-(X_{r}(t)+v_{r}(t).T_{CPA}\right)\right\|$$

Assuming that UUV encounters with a single obstacle, evaluation set of collision risk is $\left\{T_{CPA},D_{CPA}\right\}$, $\mu (D_{CPA})$ and $\mu (T_{CPA})$ are dangerous membership function $D_{CPA}$ and $T_{CPA}$. Each factor of evaluation set is defined centrally between 0 and 1.

(16) $$\mu_{t}(T_{CPA})=\{\begin{array}{ll} 0 & \left|T_{CPA}\right|>t_{2}\\ \left(\frac{t_{2}-\left|T_{CPA}\right|}{t_{2}-t_{1}}\right) & t_{1}<\left|T_{CPA}\right|\leq t_{2}\\ 1 & \left|T_{CPA}\right|\leq t_{1} \end{array}$$

(17) $$\mu_{d}(D_{CPA})=\{\begin{array}{ll} 0 & D_{CPA}>d_{2}\\ 0.5-0.5\sin\left[\frac{\pi }{d_{2}-d_{1}}(D_{CPA}-\frac{d_{1}+d_{2}}{2})\right] & d_{1}<D_{CPA}\leq d_{2}\\ 1 & D_{CPA}\leq d_{1} \end{array}$$

*t*_1_ and *t*_2_ are the time interval of arriving at closest encounter point; *d*_1_ and *d*_2_ are the distance range of arriving at closest encounter point. Each indicator has a different impact on the collision risk. With regard to UUV coarse-grained, which is acquired by weighting each indicator of collision risk, evaluation set is

(18) $$\mu_{r}=\alpha_{d}\mu_{d}(D_{CPA})+\alpha_{t}\mu_{t}(T_{CPA})$$

$\alpha_{d},\alpha_{t}(\alpha_{d}+\alpha_{t}=1)$ are the respective weighting coefficients, which can be given as $\alpha_{d}=0.35,\alpha_{t}=0.65$.

### 3.3. Screening of Key Obstacles

We introduce the concept of key obstacle for decrease the time of avoidance barriers. The key obstacle needs to satisfy three conditions. First, obstacles are within the UUV round decision range. Moreover, obstacles must be high-risk barriers (risk threshold $T_{risk}=0.5$, $\mu_{r}>T_{risk}$). In addition, UUV velocity vectors are located in the velocity barriers that be made up of obstacles. Therefore, the screening condition of key obstacles can be described as following.

(19) $$\left\{D(X_{o}^{i},X_{r})<R_{s}\right\}\wedge \left\{\mu_{r}>T_{risk}\right\}\wedge \left\{v_{r}\in VO_{i}\right\}$$

After screening, as long as one obstacle can accord with this condition, UUV must begin to prepare for avoiding obstacles.

### 3.4. The Avoidance Decision Based on the Improved Speed Barrier Method

#### 3.4.1. The Risk of Speed

**Figure 4:**
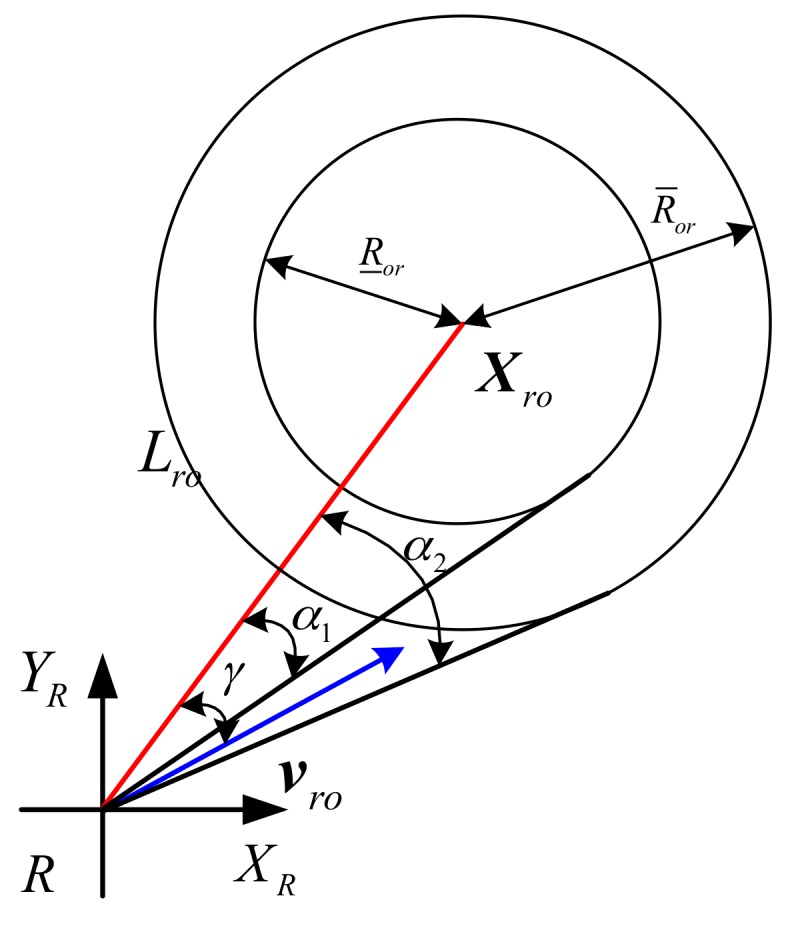
The calculation of VR.

In order to prevent collision avoidance conservative, we will associate the impact of motion uncertainty with different risk degree. As shown in Figure 4, we can gain two radius ${\underline{R}}_{or}$ and ${\bar{R}}_{or}$ after turning obstacle motion uncertainty into position uncertainty, and form two collision zones. ${\underline{R}}_{or}$ and ${\bar{R}}_{or}$ respectively are lower and upper estimate radius of obstacle $O_{i}$. ${\bar{R}}_{or}$ is determined by its location, size, speed error and other factors, here it is

(20) $$\begin{array}{l} {\underline{R}}_{or}=R_{o}+R_{r}+\delta_{p}\\ {\bar{R}}_{or}=R_{o}+R_{r}+\delta_{p}+\Delta R_{o}+R_{safe} \end{array}$$

Where $\delta_{p}$ is the estimation error of the radius of the obstacle in the obstacle classification, and $R_{safe}$ is the safe distance.

We can base on the minimum estimated radius ${\underline{R}}_{or}$ to calculate the minimum safety angle $\alpha_{1}=\arcsin(\frac{{\underline{R}}_{or}}{\left\|X_{ro}\right\|})$, and then the maximum safety angle $\alpha_{2}=\arcsin(\frac{{\bar{R}}_{or}}{\left\|X_{ro}\right\|})$. Hence, the risk of speed (VR) can be presented.

(21) $$VR_{i}(v_{ro})=\{\begin{array}{ll} 1 & \gamma \leq \alpha_{1}\\ \frac{\alpha_{2}-\gamma }{\alpha_{2}-\alpha_{1}} & \alpha_{1}<\gamma <\alpha_{2}\\ 0 & \gamma \geq \alpha_{2} \end{array}$$

Where γ is the collision angle.

When the multi-obstacles $O=\{O_{1},O_{2},\ldots ,O_{n}\}$ exist, they will produce several different speed hazards to $v_{r}$. So that the combined risk of speed for UUV can be depicted as VR.

(22) $$VR(v_{r})=1-\prod_{i=1}^{n}\left(1-VR_{i}\right)$$

#### 3.4.2. Velocity Space

Because the speed change of UUV is limited to one decision-making cycle, it cannot reflect the maximum speed space before collision of an obstacle and UUV. If collision avoidance planning is carried out in the space of single-step speed change, it is bound to cause shortsighted behavior of UUV. Predicting the velocity space can be up to within the multiple decision cycle $\Delta t_{f}(\Delta t_{f}>\Delta T)$. Considering kinematic constraints of UUV on the velocity space, the velocity space can be up to of UUV is

(23) $$\Omega_{r}=\left\{\left[\begin{array}{c} v_{x}\\ v_{y} \end{array}\right]=\left[\begin{array}{c} V_{r}\cos(\theta_{r})\\ V_{r}\sin(\theta_{r}) \end{array}\right]|\underline{V_{r}}\leq V_{r}\leq \bar{V_{r}},\underline{\theta_{r}}\leq \theta_{r}\leq \bar{\theta_{r}}\right\}$$

where $\underline{V_{r}}=\max(V_{r}^{\min},v_{r}-\Delta v_{\max}\frac{\Delta t_{f}}{\Delta T}),\bar{V_{r}}=\max(v_{r}+\Delta v_{\max}\frac{\Delta t_{f}}{\Delta T},V_{r}^{\max})$, $\bar{\theta_{r}}=\theta_{r}+\Delta w_{\max}\frac{\Delta t_{f}}{\Delta T},\underline{\theta_{r}}=\theta_{r}-\Delta w_{\max}\frac{\Delta t_{f}}{\Delta T}$, $\Delta v_{\max}$ is the maximum change in linear velocity, and $\Delta w_{\max}$ is the maximum change in yaw angular velocity in period ΔT. $V_{r}^{\max}$ is the maximum forward velocity, and $V_{r}^{\min}$ is the minimum forward velocity.

In order to reduce the complexity of calculating, its kinematic constraints can be approximately shown with maximum amplitude changes of UUV linear speed and course changes in the decision-making cycle ΔT. For prediction time of speed can be up to, the paper takes $\Delta t_{f}=4\Delta T$.

#### 3.4.3. Time to Collision

Time to collision (TTC) is the minimum time of collision between UUV and obstacles. When the relative speed of UUV and obstacles keeps unchanging, it is a common measure of collision risk evaluation, and the collision time also reflects the time limit of UUV safety. Thus, we should consider characteristics of UUV and motion characteristics of obstacles when calculating the collision time.

After expanding obstacles, considering the maximum operational range of obstacles, the obstacle velocity obstacle $VO_{i}$ is generated by the obstacle $O_{i}(X_{or},{\bar{R}}_{or})$ which radius is ${\bar{R}}_{or}$. When $v_{r}\in VO_{i}$, the collision time τ represents the shortest time of reaching the edge of the obstacle $O_{i}(X_{or},{\bar{R}}_{or})$ with the relative speed $v_{ro}$, τ satisfies the following equation

(24) $$\tau v_{ro}\in \partial (O_{i}(X_{or},{\bar{R}}_{or}))$$

Where $\partial (O_{i}(X_{or},{\bar{R}}_{or}))$ represents the edge of $O_{i}(X_{or},{\bar{R}}_{or})$. If there are many solutions about the upper formula, we can take the minimum time solution as the collision time of $v_{ro}$.

#### 3.4.4. Optimization Objective Function

Assuming that position $X_{r}$ and target position $X_{G}$ of UUV are known in any time, UUV collision avoidance in decision-making process satisfies the kinematic constraints. In view of minimum objective function of collision velocity in the decision-making cycle and speed space, dynamic collision avoidance based on the velocity obstacle method can be attributed to an optimization problem, and can be expressed as

(25) $$v^{*}=\arg\min(J_{d}(v)),\;X_{r}\to X_{G},\;v\in \Omega_{r}$$

To make UUV navigation tend to target, the target speed is defined as

(26) $$v^{ref}=V_{r}^{\max}\frac{X_{G}-X_{r}}{\left\|X_{G}-X_{r}\right\|}$$

Selection of collision avoidance speed needs to consider two factors of security and reaching the target. Therefore, optimization object function of collision avoidance decisions is defined as

(27) $$J_{d}(v)=\{\begin{array}{cc} \infty & VR(v)=1\\ \left[\omega_{p}VR(v)+\omega_{v}\frac{\left\|v-v^{ref}\right\|}{V_{r}^{\max}}\right]\times \frac{\omega_{t}}{t_{col}(v)} & \mathrm{else} \end{array}$$

The optimization object function consists of the risk of speed, the target speed deviation and the collision time. $\omega_{p},\;\omega_{v},\;\omega_{t}$ are the weight coefficients, and we can define $\omega_{p}+\omega_{v}=1$. It should be noted that when VR(v)=1, UUV sailing at v must collide with an obstacle. So v is not desirable, and $J_{d}(v)$ is given the maximum value as a punishment.

**Figure 5:**
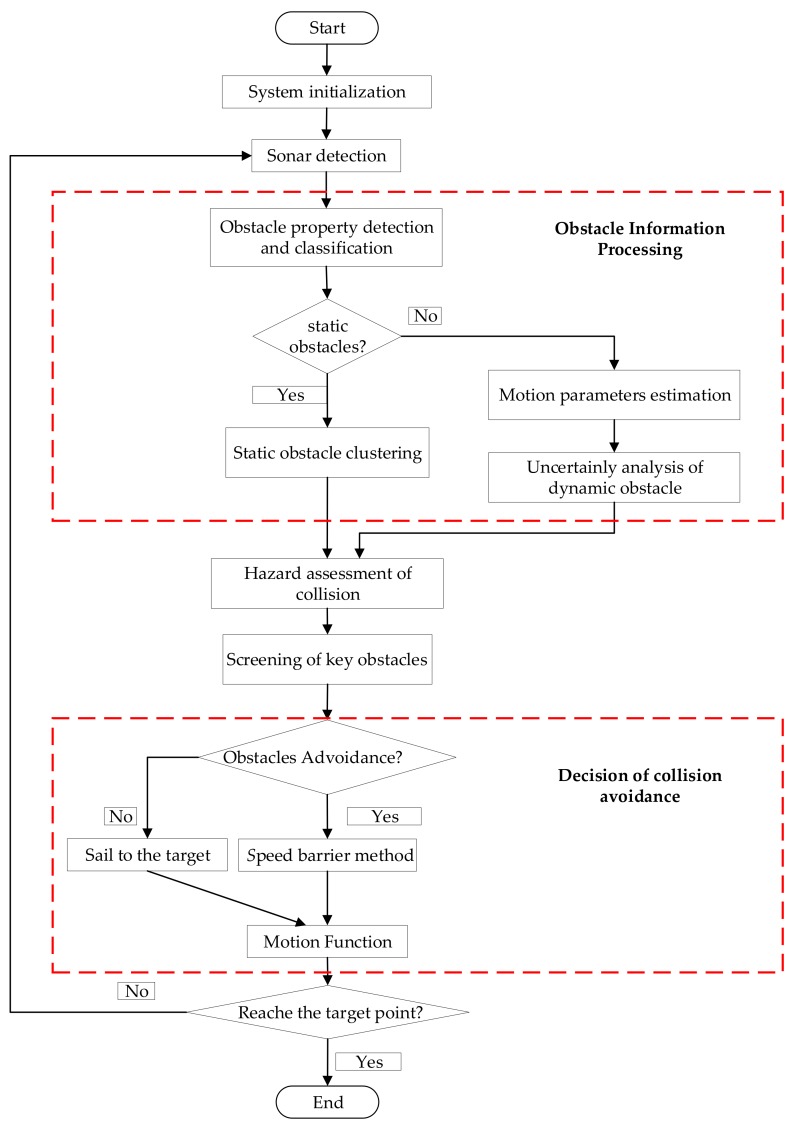
Flow chart of the dynamic obstacle avoidance.

From what has discussed above, the collision process based on the improved speed obstacle method can be presented with the flow chart of the dynamic obstacle avoidance, as shown Figure 5.

## 4. Simulations and Experimental Results

Simulations and Results are conducted to verify the effectiveness of the dynamic obstacle avoidance. As the front view sonar detection range and distance is limited, the algorithm does not recognize the moving obstacles from the rear of the UUV. In this paper, we assume that there is no obstacle coming from behind UUV. The shape of static obstacle is multi-deformation. The dynamic obstructions are considered as rectangles, wherein the diameter of the smallest circumscribed circle of the obstacle is equal to the diagonal length of the rectangle.

### 4.1. Simulation Results and Analysis

To verify the validity and accuracy of the dynamic collision avoidance method, the cases of dynamic simulation for collision avoidance are designed. Obstacle position and motion information are unknown. The motion parameters are defined as $\Delta v_{\min}=0.2\;m/s$, $V_{r}^{\max}=3\;m/s$, $V_{r}^{\min}=0.2\;m/s$ and $\Delta w_{\max}=5{}^{o}/s$. Estimation parameters of collision risk take $d_{2}=90\;m$, $d_{1}=40\;m$, $t_{2}=50\;s$, $t_{1}=30\;s$. Number of static obstacle clustering settings K is six. Optimization object function coefficients take $\omega_{p}=0.3$, $\omega_{v}=0.7$, $\omega_{t}=25$. Rate risk model parameters take $\omega_{p}=0.3$, $\omega_{v}=0.7$, $\omega_{t}=25$.

Given the initial direction and speed of dynamic obstacles, the direction and velocity of dynamic obstacles are added to the white gauss noise to simulate the random motion of dynamic obstacles. It sailed from the starting point (0, 0) to the end (450, 450). The starting heading is 45°.

**Figure 6:**
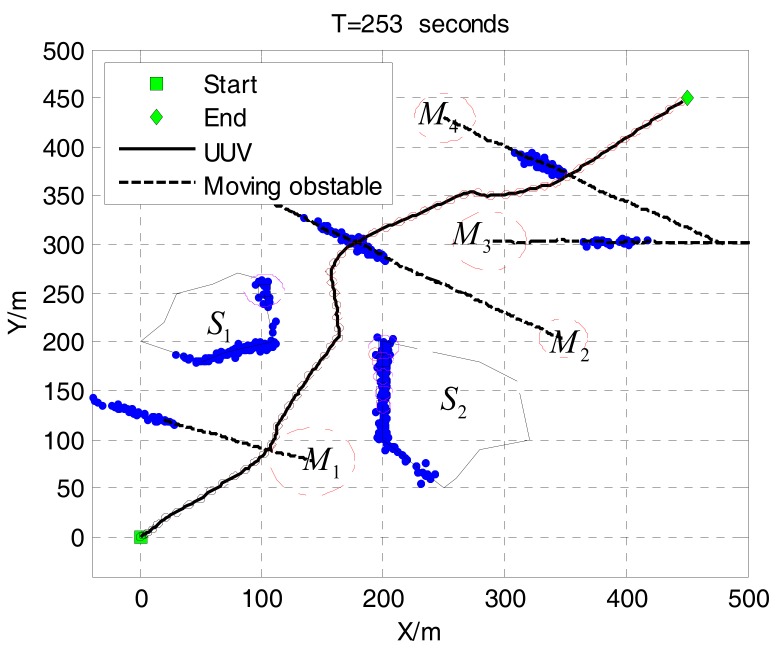
The dynamic avoidance results.

We design two polygonal static obstacles $S_{1}$, $S_{2}$ and four dynamic obstacles $M_{1},M_{2},M_{3},M_{4}$ in environment to simulate the environment of UUV voyage. The total time of the collision avoidance is 253 s, and the UUV safely arrives at the destination. Figure 6 is the environmental map and the final dynamic collision avoidance results, the figure of the circular static configuration obstacles generated by the cluster. The blue points are the obstacle information detected by the front viewer.

**Figure 7:**
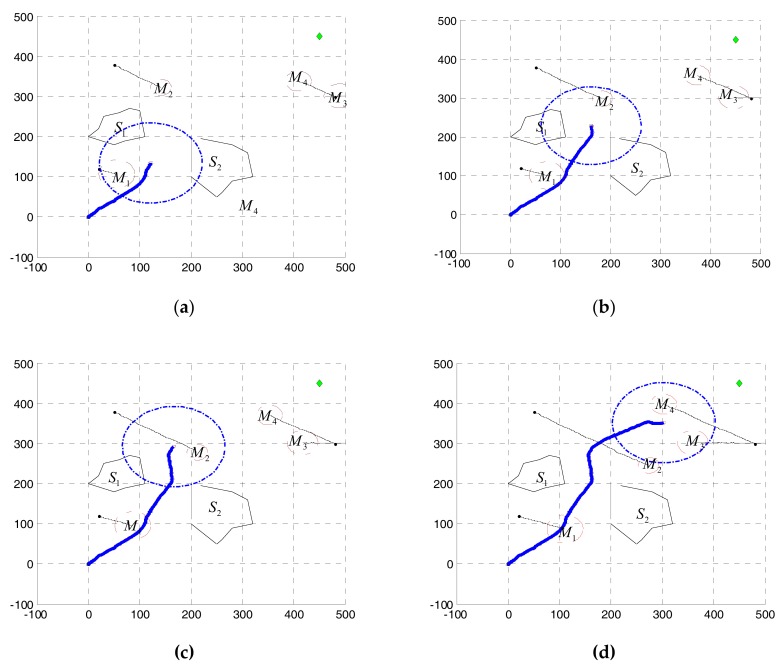
The dynamic avoidance results in different phase: (**a**) The first phase of dynamic avoidance; (**b**) The second phase of dynamic avoidance; (**c**) The third phase of dynamic avoidance; (**d**) The fourth phase of dynamic avoidance.

**Figure 8:**
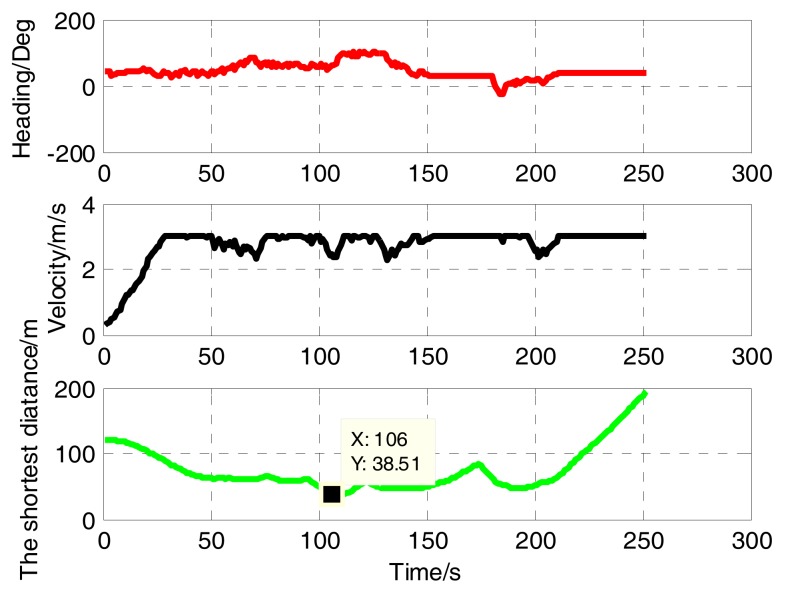
The heading, velocity and the shortest distance.

Figure 6 and Figure 7 are based on the same environment map, which shows the process of dynamic obstacle avoidance. The distribution of obstacles and the effect of avoidance can be found from this figure. The blue circle represents the UUV detection range. In Figure 7a, UUV finds the dynamic obstacle $M_{1}$, turns left and avoids successfully. Then it found six static obstacles, turned slightly left, and through that small space safely in Figure 7b. Obviously, the six smaller static obstacles are produced by clustering two large static obstacles. Similarly, Figure 7c shows that the dynamic obstacle $M_{2}$ is successfully discovered and avoided. In Figure 7d, UUV find the obstacles $M_{3},M_{4}$ at the same time, and the $M_{4}$ is considered to be the key obstacle after the decision. Finally, UUV reaches the target point. Figure 8 shows the UUV heading, speed and the shortest distance with obstructions in this process. Throughout the process, UUV heading and speed changes are smaller, we can see that the proposed method was conducted well. The minimum distance between UUV and obstacles is 38.51 m, which can fully guarantee the safety of UUV.

### 4.2. Experimental Results and Analysis

**Figure 9:**
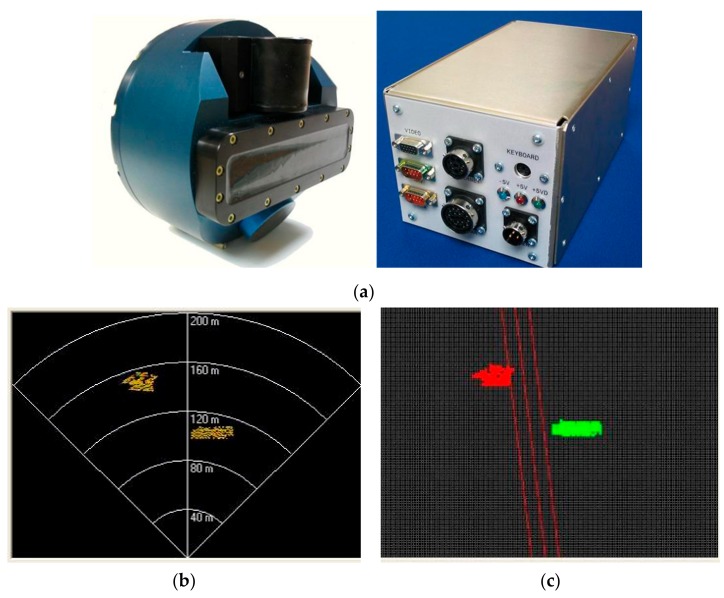
Expression sonar image by occupancy grid: (**a**) Sonar sensor and PC104 processor; (**b**) Sonar images of object; (**c**) the grid figure.

In order to test the adaptability of the method in the unknown dynamic environment, we design the following experiment. To get the data to detect obstacles, the sensing device uses the multi-beam forward-looking sonar, as shown in Figure 9a. The multi-beam forward-looking sonar has 60 ceramic receivers, the open angle is 90°, vertical open angle is 6° or 12°, angular resolution of sonar is 1.69°, the maximum detection range is 200 m.

The multi-beam forward-looking sonar uses the occupancy grid to represent the local environment. Sonar images of the object are shown in Figure 9b, the object’s color represents the strength of the signal. Sonar images are collected to be discretized into the grid in every moment, as shown in Figure 9c. In terms of accuracy and calculation, the size/resolution of the grid is selected as 5 m × 5 m. UUV can easily extract the various parameters of the obstacle to avoid obstacles by using the occupancy grid.

**Figure 10:**
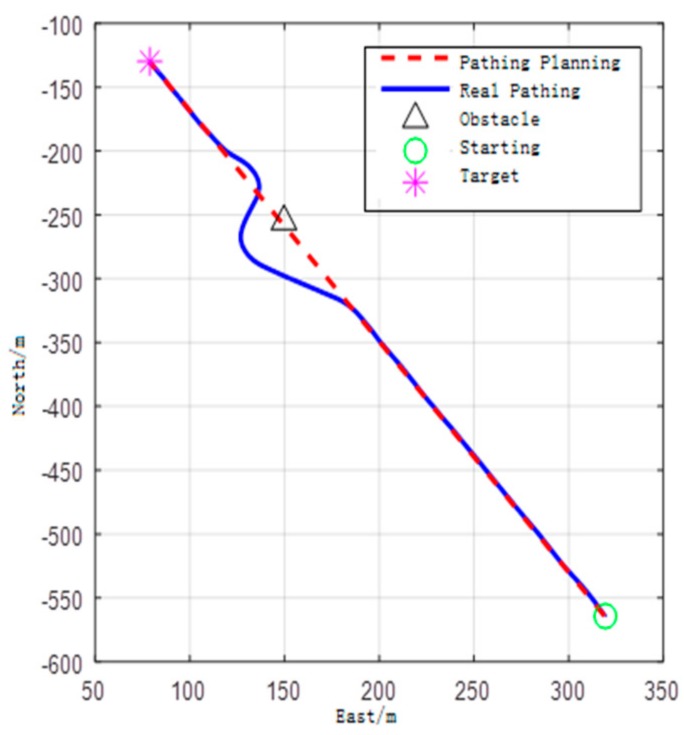
The dynamic avoidance results.

Based on sonar information processing capabilities, we designed a static obstacle avoidance experiment based on the proposed algorithm. First, the UUV determined that it was a dynamic obstacle of zero velocity (static obstacle) by processing the data obtained by sonar and classified the static obstacle into several small landmark obstacles by clustering. Then UUV used the improved speed obstacle method to avoid obstacles in time. The experimental results are shown in Figure 10. In the local NED coordinate system, selecting a point as the origin to establish relative coordinates, north sets as Y-axis, east sets as X-axis. It sailed from the starting point (80, −125) to the end (320, −575). A static obstacle was located in point (150, −250). The starting heading is 135°.

The blue line represents the shortest path of UUV, the red dotted line is the path in offline planning. In Figure 10, UUV sailed according to the direction of the shortest path; when UUV detects obstacles, UUV gets accurately around the obstacle to target. Due to the interference of temperature gradient, surface ware, sonar carrier motion and so on, there is a certain virtual image rate in sonar, which makes collision avoidance decision time greatly prolonged. The entire avoidance decision-making process takes 1–2 s, and the occupied computer memory is about 2 MB. The introduction of risk of speed expansion strategy can eliminate the problem of conservative collision avoidance of direct expansion of the dynamic obstacles, and improve the efficiency of collision avoidance.

## 5. Discussion

Through the above simulations and experiment, we can prove the following conclusions:

1. The introduction of collision risk and screening key obstacles can obtain the right moment to avoid collision.
2. Large-scale static obstacle clustering treatment and common identification of moving and static barriers can reduce the complexity of dynamic collision avoidance, and effectively avoid large static obstacles.
3. Based on the speed risk, the puffing strategy can solve the conservative collision avoidance problems caused by the direct expansion of obstacles.

In the future, on the one hand, we will conduct a more comprehensive analysis of the dynamic obstacle avoidance process, making it suitable for a variety of complex environments such as waves and undercurrent. On the other hand, we will put forward a dynamic obstacle avoidance method based on improving speed obstacles in practice. By replacing more accurate multi-beam forward-looking sonar and faster processors, our algorithm can be used in a real marine environment.

## 6. Conclusions

This paper proposes the dynamic collision avoidance method based on improved velocity obstacles, and the real-time collision avoidance problem under the dynamic obstacle environment can be solved. Aiming at the existing problem of velocity obstacle method, this method is improved. According to DCPA and TCPA requirement, we establish a collision risk evaluation model, and combine with the discriminant conditions of velocity obstacle. Then, we can get the right timing of collision avoidance, reduce the computing burden of collision avoidance decision-making and improve the speed of collision avoidance. The motion uncertainty of obstacles and velocity obstacles is considered. The collision impact brought by obstacle motion uncertainty is reduced, and the conservative problem of dynamic obstacle collision avoidance brought by direct extrusion can be avoided. The comprehensive optimization objective function of speed risk degree, the target speed deviation and the collision time is designed, and it can dramaticly improve the security of collision avoidance. Meanwhile, the UUV can reach to the target location as soon as possible. Finally, the simulation results show that the proposed method has quick speed of decision-making for collision avoidance, it can avoid all kinds of obstacles better under a dynamic environment, and has a good adaptability to the unknown dynamic environment.
